# Dissecting genetic factors affecting phenylephrine infusion rates during anesthesia: a genome-wide association study employing EHR data

**DOI:** 10.1186/s12916-019-1405-7

**Published:** 2019-08-28

**Authors:** Yanfei Zhang, S. Mark Poler, Jiang Li, Vida Abedi, Sarah A. Pendergrass, Marc S. Williams, Ming Ta Michael Lee

**Affiliations:** 1 Genomic Medicine Institute, Geisinger, Danville, PA 17822 USA; 2 Department of Anesthesiology, Geisinger, Danville, PA 17822 USA; 3 Biomedical Translational Informatics Institute, Geisinger, Danville, PA 17822 USA; 4 Biomedical Translational Informatics Institute, Geisinger, Bethesda, MD USA; 5 Lab 218, Weis Center for Research, Geisinger, 100 North Academy Ave, Danville, 17822-2620 PA USA

**Keywords:** Phenylephrine, Anesthesia, Hypotension, GWAS, *EDN2*, Electronic health records (EHR), Unsupervised clustering, Vasoconstriction, Pharmacogenomics

## Abstract

**Background:**

The alpha-adrenergic agonist phenylephrine is often used to treat hypotension during anesthesia. In clinical situations, low blood pressure may require prompt intervention by intravenous bolus or infusion. Differences in responsiveness to phenylephrine treatment are commonly observed in clinical practice. Candidate gene studies indicate genetic variants may contribute to this variable response.

**Methods:**

Pharmacological and physiological data were retrospectively extracted from routine clinical anesthetic records. Response to phenylephrine boluses could not be reliably assessed, so infusion rates were used for analysis. Unsupervised *k*-means clustering was conducted on clean data containing 4130 patients based on phenylephrine infusion rate and blood pressure parameters, to identify potential phenotypic subtypes. Genome-wide association studies (GWAS) were performed against average infusion rates in two cohorts: phase I (*n* = 1205) and phase II (*n* = 329). Top genetic variants identified from the meta-analysis were further examined to see if they could differentiate subgroups identified by *k*-means clustering.

**Results:**

Three subgroups of patients with different response to phenylephrine were clustered and characterized: resistant (high infusion rate yet low mean systolic blood pressure (SBP)), intermediate (low infusion rate and low SBP), and sensitive (low infusion rate with high SBP). Differences among clusters were tabulated to assess for possible confounding influences. Comorbidity hierarchical clustering showed the resistant group had a higher prevalence of confounding factors than the intermediate and sensitive groups although overall prevalence is below 6%. Three loci with *P* < 1 × 10^−6^ were associated with phenylephrine infusion rate. Only rs11572377 with *P* = 6.09 × 10^−7^, a 3′UTR variant of *EDN2*, encoding a secretory vasoconstricting peptide, could significantly differentiate resistant from sensitive groups (*P* = 0.015 and 0.018 for phase I and phase II) or resistant from pooled sensitive and intermediate groups (*P* = 0.047 and 0.018).

**Conclusions:**

Retrospective analysis of electronic anesthetic records data coupled with the genetic data identified genetic variants contributing to variable sensitivity to phenylephrine infusion during anesthesia. Although the identified top gene, *EDN2*, has robust biological relevance to vasoconstriction by binding to endothelin type A (ET_A_) receptors on arterial smooth muscle cells, further functional as well as replication studies are necessary to confirm this association.

**Electronic supplementary material:**

The online version of this article (10.1186/s12916-019-1405-7) contains supplementary material, which is available to authorized users.

## Background

Phenylephrine is a very selective α_1_-adrenergic receptor agonist frequently used for treatment and prevention of hypotension during anesthesia or critical care. It is one of the most commonly used drugs for treatment of intraoperative hypotension [1]. Phenylephrine infusions are used to sustain blood pressure at clinically acceptable levels during anesthesia. Inter-individual differences in response to phenylephrine have been frequently observed in clinical practice [2–4]. Patients’ response to phenylephrine may vary depending upon age, co-morbidities (e.g., cardiovascular diseases), concurrent medications, and anesthetic status. Genetic factors may also contribute to this response variability.

While some work has been done looking at genetic influence of *ADRB2* on hemodynamic response [5, 6], very little work on phenylephrine response has been published beyond some limited candidate gene studies. Although it functions as a selective α_1_-adrenergic receptor agonist, phenylephrine has a moderate β-agonist activity at higher doses [7, 8]. Several candidate gene studies have found that individuals carrying the Ile164 allele in *ADRB2*, encoding the β_2_ adrenoreceptor, had a much higher sensitivity to phenylephrine than non-carriers [9]. The Arg16 allele alone or Arg16-Gln27-Thr164-Arg175-Gly351 haplotype was also associated with higher phenylephrine sensitivity [10]. However, this association could not be replicated in a cohort of patients under spinal anesthesia for cesarean delivery and the Arg16 carriers actually required more phenylephrine than non-carriers [11]. The association between 34 single nucleotide polymorphisms (SNP) in *ADRA1B*, encoding α_1_ adrenergic receptor 1B subtype, and phenylephrine response was evaluated [12]. rs10070745 was significantly associated with response to this vasoconstrictor only in patients with African but not European ancestry [12]. There was no report of significant impact of genetic variants from *ADRA1A*, encoding α_1_ adrenergic receptor 1A subtype, on phenylephrine response. Although all these candidate gene studies showed some promising connection between pharmacodynamic genes and drug response, the significance of the association has been limited by sample size, number of interrogated genetic variants, definition of responsiveness (response or non-response as a binary trait), magnitude of variation in response (quantitative trait), mixture of vasopressors, and hypotheses. Genome-wide association studies (GWAS) employ large patient cohorts and subsequent fine-mapping techniques that are hypothesis-agnostic and thus not limited to preconceived ideas of the genes involved in the phenotype based on prior knowledge.

In clinical situations, phenylephrine is most often administered as boluses, less often by infusion. An immediate increase of BP after a bolus, usually prompt in onset and lasting for minutes, could be used to evaluate responsiveness to phenylephrine. This requires frequent BP measurements during the effect window, a requirement not satisfied by the available research data extracted from clinical anesthesia records. Alternatively, intravenous (IV) infusion rate (dose) could be used to estimate phenylephrine sensitivity. Infusion rates are determined empirically by clinicians, adjusted dynamically as needed by changing conditions, and subject to numerous unaccounted determinants (e.g., blood loss and volume replacement, concurrent drug effects, and surgical events and requirements) to maintain situationally appropriate blood pressures. Infusion rates can be quite variable while being titrated to effect, then rather stable for long periods after completion of initial adjustments. With a large patient cohort, individual variations are expected to be randomly distributed relative to the genetic variations.

Geisinger is an integrated healthcare provider located in central and northeastern Pennsylvania and southern New Jersey, having an electronic health record (EHR) system which captures a median of 14 years of comprehensive electronic records for participants in the MyCode® Community Health Initiative (MyCode) which include but are not limited to patients’ demographic features, primary diagnoses and co-morbidities, laboratory measurements, prescriptions, vital signs, and surgical procedure logs [13]. Intraoperative electronic anesthesia records have been active since July 2012. Whole exome sequencing and genome-wide genotyping data are available for more than 92,000 MyCode participants to date [13, 14]. The coupled genotype and longitudinal phenotype data provide unique opportunities for us to conduct GWAS based on this “real world” clinical data and to yield clinically relevant insights [15].

In this study, we present the results of the first GWAS for phenylephrine response defined by phenylephrine infusion rate using real-world EHR data.

## Methods

### Study cohort and institutional review board

This study population consisted of 12,688 individuals with available electronic anesthesia records from the Geisinger de-identified EHR database who met the inclusion criteria described in more detail below. We received an exemption from the institutional review board (IRB) for a non-human subject study as all the EHR data were de-identified. For the genetic study, we received approval from the IRB at Geisinger and the MyCode Governing Board. All MyCode participants provide a consent that allows their clinical and genomic data to be used for health-related research. Details of the consenting process are described elsewhere [9]. Approximately 40% of these patients with available anesthesia records were MyCode participants with genetic data.

### EHR data extraction

In Geisinger clinical practice, as at many other institutions, phenylephrine is typically the first-line vasopressor for treatment of hypotension during anesthesia. However, there are no standardized phenylephrine infusion guidelines, BP targets, or practices for cases in this opportunistic cohort. Pharmacy prepares standard concentrations of phenylephrine for infusions. Choice and management of phenylephrine infusions was entirely at the discretion of anesthesia clinicians. General anesthesia predominates for surgeries at Geisinger, although spinal and regional anesthetics are employed with and without general anesthesia. Data de-identification and extraction were conducted by Geisinger’s Phenomic Analytics & Clinical Data Core. Patient information of those who had electronic anesthesia records between July 2012 and November 2016 excluding cesarean sections and trauma cases was obtained. The following de-identified data elements were requested: surgery duration; anesthetic agents and other intraoperative drugs, doses and total quantity of phenylephrine; start and end time of each phenylephrine infusion; phenylephrine infusion rates; blood pressures; age and weight at the time of surgery; International Classification of Disease v. 9 (ICD-9) coded diagnoses; and demographics. The extracted anesthetic record data originated from multiple Geisinger outpatient and inpatient sites distributed across a large geographical area. There was no selection for patient characteristics, types of procedures, surgical departments, or anesthetic techniques.

### EHR data mining and modeling

#### Quality control (QC) and sample filtration

**Fig. 1:**
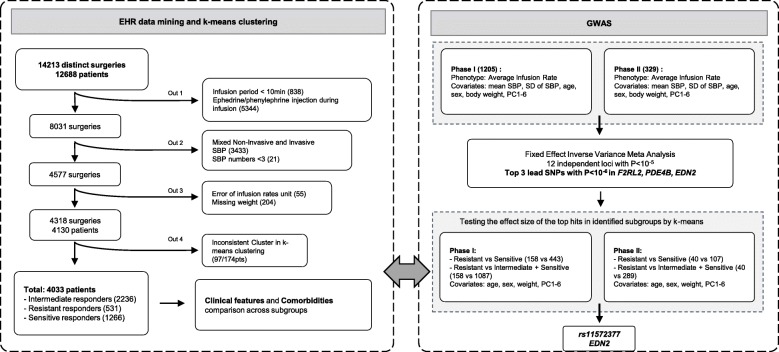
EHR data mining and GWAS pipeline. A total of 14,213 distinct anesthesia episodes that had phenylephrine infusions were identified from the EHR data. 4033 patients were included for *k*-means clustering after applying exclusion criteria such as a short infusion period (< 10 min), concurrent ephedrine and/or phenylephrine bolus injections during the infusion period, blood pressure values that were not consistently obtained by either invasive or non-invasive measurement, a limited number of SBP measurements, implausible infusion rate units, and missing body weight. Linear regression model for phenylephrine infusion rate was selected for association testing under an additive genetic mode followed by a fix effect inverse variance meta-analyses of phase I and phase II datasets. Top associated loci were further tested by comparing resistant versus sensitive or resistant versus pooled sensitive and intermediate groups. SBP, systolic blood pressure

Analyses employed SBP because it is the most consistently present blood pressure parameter and has larger changes in response to phenylephrine treatment than mean artery pressure (MAP) or DBP, though MAP may be a more robust physiological measurement and better correlated between invasive and non-invasive BP measurements. We removed obvious errors in the data by identifying values that were not representative of the measurement, such as implausible values (e.g., BMI = 1000), and extreme SBP values (SBP < 20 mmHg or SBP > 200 mmHg) as they were less likely to be caused by genetic factors, but more likely to be data errors or artifacts, such as arterial line flushes, disconnections, or physiological extremis. Patients who had ephedrine and/or phenylephrine boluses during phenylephrine infusion, patients who had short infusion periods (< 10 min), or few blood pressure measurements during the infusion interval (< 3 data points) were also excluded from further analysis. Median count for SBP measurements per patient is 16. These quality-checked data were then used for data modeling (Fig. 1).

#### Data modeling

Empirical observation discerns three types of response to phenylephrine infusion: normal or high BP with low infusion rate (“sensitive”), correction of severe hypotension but still low BP with low infusion rate (“intermediate”), and ameliorated yet still low BP requiring high infusion rate (“resistant”). A “fourth” hypothetical cluster having high BP at high infusion rates is not represented in the data because clinicians rarely need to use phenylephrine to increase already normal or high blood pressures. *k*-means clustering was selected as our unsupervised machine learning algorithm as it is simple and computationally efficient to solve known cluster issues within large datasets. To reflect the clinical observations, cluster numbers (*k* = 2 and 3) were evaluated using three key features related to phenylephrine response: mean and standard deviation (SD) of systolic blood pressure (SBP) during infusion period and average infusion rate of phenylephrine. We also evaluated the *k*-means clustering on a broader set of features including weight, age, and infusion duration. Data standardization and *k*-means clustering were performed using Python (2.7.14) scikit-learn library (0.19.1).

#### Clinical feature analysis

To determine whether there were statistical differences in clinical features between clustering derived subgroups, chi-square tests were used for categorical data, and one-way ANOVA was used for continuous data. These statistical analyses were performed using GraphPad Prism (7.04). The *P* value < 0.00625 (0.05/8) was considered significant after the Bonferroni correction for multiple comparisons.

#### Comorbidity analysis

ICD-9 codes were used at the 3-digit level. For example, individuals with the codes 203.01 (multiple myeloma, in remission) and/or 203.12 (plasma cell leukemia, in relapse) would be evaluated in the same group 203 (multiple myeloma and immunoproliferative neoplasms). Patients with the same truncated 3-digit codes on at least three different dates were considered to have reportable evidence for the 3-digit disease code class. Diseases with prevalence ≥ 0.5% in all the three groups were clustered via hierarchical clustering using Euclidean distance and average linkage and plotted in a heatmap using R (version 3.4.3). Chi-square test was used to evaluate the significance of the difference among subgroups.

### Genotyping and imputation

Genotyping was performed in batches on the Illumina Infinium OmniExpress Exome array and GSA-24v1-0 array at different time for phase I and phase II cohorts, respectively. Genotypes for both cohorts were imputed to HRC.r1-1 EUR reference genome (GRCh37 build) separately using the Michigan Imputation Server [16]. Variants with an info score > 0.7 were included in the analysis. Samples with genotyping rate below 95% were excluded. SNPs with < 99% call rate, minor allele frequency < 1%, and significant deviation from Hardy-Weinberg equilibrium (*P* < 10^−7^) were removed. At last, there were 4,929,806 SNPs in phase I and 2,978,370 SNPs in phase II included in the analysis. One of paired of individuals with first- or second-degree relatedness, as determined by IBD analysis were removed from analysis. PLINK 1.9 [17] was used for genotypic quality control.

### Association tests and the meta-analysis

The flowchart and sample size of phase I and phase II cohorts are shown in Fig. 1. Ninety-seven of the 174 patients, who had multiple surgeries partitioned to different clusters for different procedures by *k*-means clustering, were removed from further analyses. The mean values of the SBP, age, and weight for the other 77 patients who had concordant cluster associations from multiple surgeries were used to avoid non-independent measurements in the association test. Finally, 1574 patients were subject to genetic analyses. A linear regression model for average infusion rate with full set of covariates without interaction terms was conducted first to decide which covariates to adjust in the genetic association test. Covariates that were evaluated included age, sex, weight, mean SBP, SD of SBP, anesthesia type, and comorbidities that may affect the pharmacokinetics and blood pressure, including diabetes, hypertension, lipid metabolism disorders, overweight and obesity, ischemic heart disease, heart failure, and cardiac dysrhythmias. Only age, sex, weight, mean of SBP, and SD of SBP were significantly associated with the infusion rate (Additional file 1: Table S1). Thus, they were included together with first six principal components (PCs) as covariates in GWAS tests.

PLINK 1.9 was used to perform the genetic association analyses. A linear regression model was adopted for GWAS on the average infusion rate adjusted for the significant covariates and the first six principal components in phase I and phase II cohorts followed by a fixed effect inverse variance meta-analysis by METAL [18]. The lead SNPs were further evaluated in the case-control association test in subgroups identified by *k*-means clustering using logistic models adjusting for age, sex, body weight, and 6 PCs. Mean and SD of BP were used in the determination of clusters and thus were not included in the model. The resistant group was compared to the sensitive group alone, or the pooled intermediate + sensitive groups.

GTEx [19], Ensemble VEP [20], USCS genome browser [21], and STRING [22] were used for eQTL, variant annotation, and other functional genomics queries. Power test for the top hit with strong biological relevance was conducted using Quanto.

## Results

A total of 14,213 distinct anesthesia episodes that had phenylephrine infusions were identified from the EHR data. Of these, 9895 were excluded during data cleaning due to a short infusion period (< 10 min), concurrent confounding ephedrine and/or phenylephrine bolus injections during the infusion period, blood pressure values that were not exclusively obtained by either invasive or non-invasive measurements, fewer than three SBP measurements during phenylephrine infusion, implausible infusion rate units, and missing body weight (Fig. 1). A total of 4033 unique patients were included in the analyses. The demographic features are listed in Table 1. The average age at the time of surgery was 62.4 years old. Most of the patients are of European ancestry (97.7%) and had surgeries performed under general anesthesia.

**Table 1:** Population demographics

Characteristics	Value
Total number of patients	4033
Sex (M, F)	1902, 2131
Age, years (mean ± SD)	62.4 ± 14.4
Race	
European ancestry	3941
African ancestry	70
Other	22
Anesthesia type	
General	3709
Spinal	163
Others	161

### Three sub-groups were identified for phenylephrine responsiveness

**Fig. 2:**
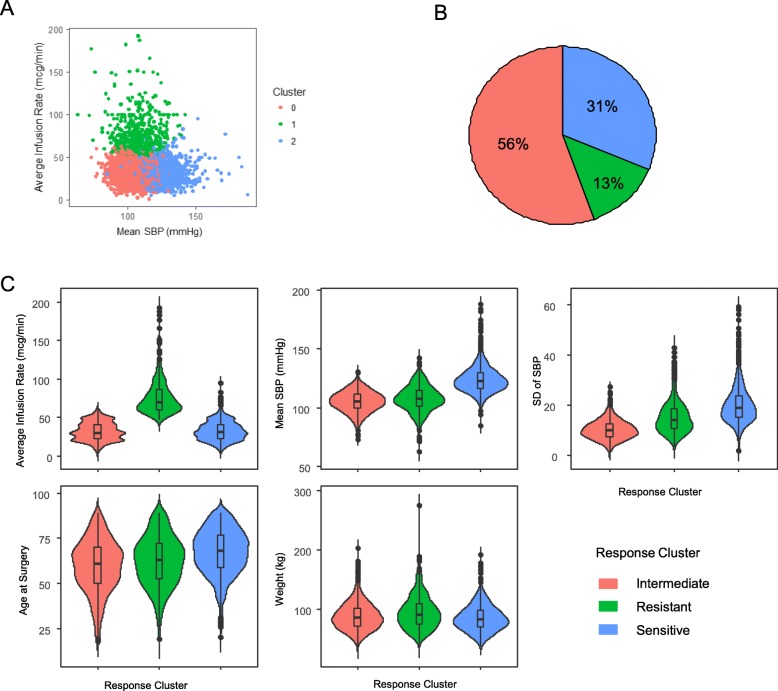
*k*-means clustering based on phenylephrine infusion rate, mean SBP, and SD values of SBP. **a** Scatter plot of mean SBP against average infusion rate after *k*-means clustering when *k* = 3. **b** Pie chart to show the proportion of each identified sub-group. **c** Violin plots to show the distribution of individual clinical parameter among three clustered subgroups. *X*-axis represents the response clusters: resistant, intermediate, and sensitive. *Y*-axis represents each clinical parameter. SBP, systolic blood pressure; SD, standard deviation

*k*-means clustering was employed as our unsupervised machine learning approach to categorize the response to phenylephrine, for 2 or 3 clusters evaluated on three key features related to phenylephrine response: average infusion rate (mcg/min), mean SBP (mmHg), and SD of SBP. SD of SBP reflects the blood pressure variability during the infusion period. When two clusters were considered (*k* = 2), the separation was made by blood pressures but not by average infusion rate (Additional file 2: Figure S1A). In a two-cluster model, 36% of the patients were classified as poor responders (Additional file 2: Figure S1B), which was higher than clinically observed empirical rate of 10~20%. When three clusters were evaluated (*k* = 3), patients were classified into three categories that can be described as *intermediate responders (n = 2236, 56%)*, having low mean SBP under low infusion rate of phenylephrine; *resistant responders (n = 531, 13%)*, requiring higher infusion rate to maintain low mean SBP; and *sensitive responders (n = 1266, 31%)*, having higher mean SBP with low infusion rates (Fig. 2a and b). Ninety-seven patients were dropped from the analysis due to inconsistent clustering when comparing the results from two or more anesthesia episodes for the same individual. There were no clear boundaries between clusters when average infusion rate was plotted against mean SBP (Fig. 2a), indicating the response is not a discrete trait; however, the degree of overlap is modest, supporting the clinical observations.

We have evaluated *k*-means clustering on other feature combinations. However, we did not observe more definitive or distinct clusters. In contrast, the boundaries became less definitive with the number of features increasing, especially for the resistant group, which is the most clinically interesting (Additional file 3 Figure S2).

### Clinical features among sub-groups of distinct phenylephrine responsiveness

Since responsiveness can be affected by multiple genetic or non-genetic factors, the clinical features in each subgroup were further compared by chi-square test or one-way ANOVA for categorical and quantitative features, respectively (Fig. 2c and Table 2). The means for average infusion rate, SBP, and SD of SBP were significantly different among three clusters. Other than anesthesia type, clinical features such as age, sex, and weight, were significantly different among these groups (Table 2). The resistant group had a lower percentage of female patients (*P* = 2.17 × 10^−10^) and higher body weight (*P* = 0.0016) than the intermediate and the sensitive groups, suggesting that sex and body weight could possibly be predictors of phenylephrine response and should be included as confounding factors in the association study. One of the key pharmacokinetic parameters, volume of distribution (VD) of a drug, which is the circulation volume for phenylephrine infusion, could be significantly affected by body weight. The mean age of sensitive responders was significantly higher than that of intermediate (*P* = 1.759 × 10^−12^) and resistant patients (*P* = 2.2 × 10^−16^).

**Table 2:** The clinical features corresponding to the three groups

	Resistant	Intermediate	Sensitive	*P* value*
No. of Patients	531	2236	1266	/
Sex: female, *N* (%)	239 (45.0)	1131 (50.6)	761 (60.1)	< 0.0001
Anesthesia type %				
General	421 (79.3)	1806 (80.9)	1068 (84.3)	
Spinal	21 (3.9)	99 (4.4)	43 (3.4)	0.0354
Others	89 (16.8)	331 (14.7)	155 (12.3)	
Age at surgery (years)	62.4 ± 13.9	59.5 ± 14.7	67.4 ± 12.5	< 0.0001
Body weight (kg)	94.7 ± 27.6	88.6 ± 23.0	85.4 ± 22.1	< 0.0001
Avg. infusion rate (mcg/min)	76.2 ± 22.4	32.1 ± 11.8	32.4 ± 12.3	< 0.0001
Mean SBP (mmHg)	108.0 ± 11.0	105.4 ± 8.7	123.9 ± 10.9	< 0.0001
SD of SBP	15.0 ± 6.4	10.0 ± 3.7	19.9 ± 7.3	< 0.0001

*P value was calculated from chi-square test for categorical variables, and *one-way ANOVA* for continuous variables. *P value* < 0.00625 was considered significant

### Different comorbidities among sub-groups of phenylephrine responsiveness

**Fig. 3:**
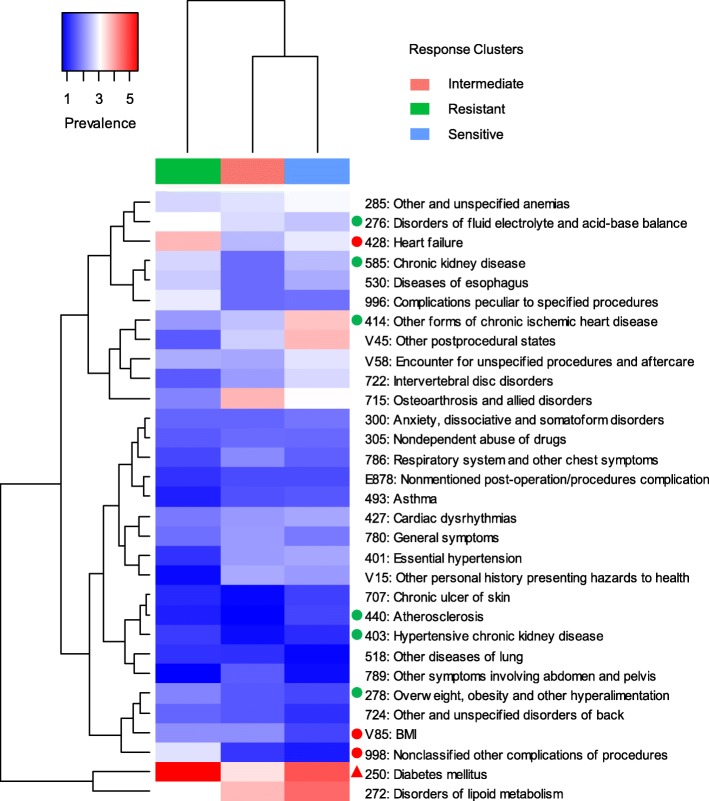
Heatmap of comorbidity prevalence in the three subgroups identified by *k*-means clustering. *X*-axis represents the subgroups; *Y*-axis represents the comorbidities. Hierarchical clustering was performed on both *X*- and *Y*-axis using Euclidean distance and average linkage method. The redder, the higher prevalence. The greener, the lower prevalence. The prevalence of disease across the subgroups was evaluated by chi-square test. The red triangle represents *P* < 0.001; the red dot represents *P* < 0.01; the green dot represents *P* < 0.05

Comorbidities and the three subgroups of patients with differential response to phenylephrine were clustered and characterized. The resistant group was well separated from the sensitive and intermediate groups in hierarchical clustering (Fig. 3). The resistant group has a higher prevalence of diabetes (*P* = 0.00023), heart failure (*P* = 0.003), chronic kidney disease (*P* = 0.02), overweight (*P* = 0.04), and disorders of fluid-electrolyte and acid-base balance (*P* = 0.04), suggesting more confounding factors and severity of illness in the resistant group. These could reflect physiological alterations or more clinically significant compromise in these patients, requiring more aggressive treatment of hypotension. The overall calculated comorbidity prevalence was low (< 6%) across all three subgroups. This might explain why comorbidities were not significant covariates in the full model on phenylephrine infusion rate (Additional file 1: Table S1).

### Top associations with phenylephrine infusion rate

**Fig. 4:**
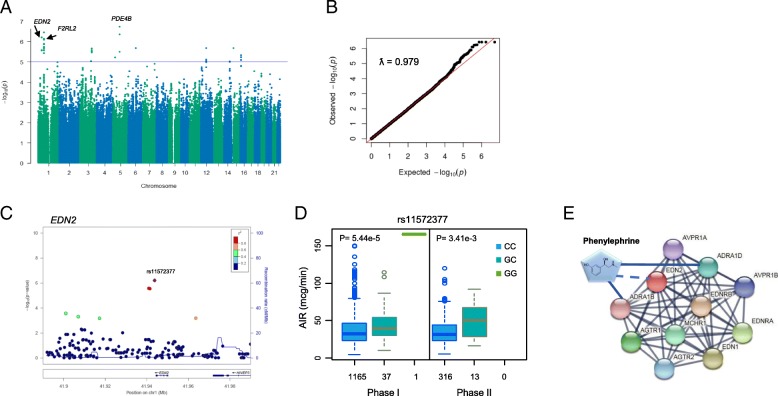
Meta-analysis and subsequent analyses on top hits. **a** Manhattan plot and **b** Q-Q plot of the meta-analysis for phenylephrine average infusion rate. Top loci with *P* < 10^–6^ were labeled. Genome inflation factor ƛ was 0.979. **c** Regional association for *EDN2* in meta-analyses for phenylephrine infusion rate. 800 kb flanking the genomic region of the lead SNP, marked as purple diamond, were illustrated. **d** Boxplot of average infusion rate against the rs11572377 genotypes in phase I and phase II cohorts. Raw *P* value refers to allelic association under additive model of linear regression adjusted for the corresponding covariates. **e** Protein-protein interaction network for EDN2 identified by STRING. The nodes and edges respectively represent encoded proteins and evidence-based functional interaction derived from a combined score which was computed by combining the probabilities from up to 7 different resources and corrected for the probability of randomly observing an interaction. Only high confidence interactions were shown here (interaction score ≥ 0.9). Phenylephrine node was superimposed to the existing interactive plot. A solid line was created between phenylephrine and ADRA1B or phenylephrine and ADRA1D because of converging evidence from literature. A dashed line was created to show the hypothetical connect between phenylephrine and EDN2 due to lack of solid evidence from literature

All patients included in this GWAS have genetically verified European ancestry, a characteristic of the Geisinger regional population that may not be generalizable. We applied a linear regression model to identify the potential confounding factors related to average infusion rate (Additional file 1: Table S1). Patients’ age at surgery, sex, and body weight were considered as the covariates in all subsequent association testing. No genome-wide significant loci were identified in either the phase I or phase II cohorts. Twelve independent loci with suggestive significance (*P* < 10^−5^) were identified to be associated with the phenylephrine average infusion rate after a meta-analysis. Figure 4a and b illustrate the Manhattan plot and QQ plot for the meta-analysis. Table 3 lists the lead SNP in each locus after a LD-based clumping of the summary statistics. The most significantly associated SNP was rs2069661, flanking the bidirectional genes, *F2RL2* and *IQGAP2*. This SNP is in complete linkage disequilibrium (LD) with rs193230021 and rs116836657, both of which are located at 3′UTR for *F2RL2*. rs77080086, the second top hit, is an intronic variant in *PDE4B* that encodes an enzyme that specifically hydrolyzes cAMP, a critical step in the β-adrenergic receptors signaling [23]. The lead SNP is in complete LD with rs75398902 (A112G), a missense SNP, which is possibly damaging as predicted by PolyPhen (score of 0.506). This mutation may affect the alpha-helix stability of the functional domain of the enzyme [24]. The third top SNP, rs11572377, located at the 3′UTR of *EDN2*, encodes endothelin-2, a secretory vasoconstrictive peptide which causes vasoconstrictions by tightly binding to smooth muscle ET_A_ receptors [25].

**Table 3:** Summary of lead SNP (LD clumped) that show suggestive associations (*P* < 10^−5^) with phenylephrine average infusion rate in a meta-analysis

Lead SNP	Coordinate (GRCh37)	A1/A2	MAF	Beta-meta (CI95)	P-PhaseI	P-PhaseII	P-meta	Gene	Upstream gene	Downstream gene
rs2069661	5:75916615	A/C	0.01725	13.85 (8.65~19.04)	5.71E−06	1.15E−02	1.80E−07	*F2RL2*, *IQGAP2*	*LOC101929109*	*NCRUPAR*
rs77080086	1:66727250	T/C	0.02468	11.07 (6.81 ~ 15.33)	5.50E−07	1.97E−01	3.51E−07	*PDE4B*	*LOC101927139*	*LOC105378776*
rs11572377	1:41943795	C/G	0.01689	13.00 (7.89 ~ 18.11)	5.44E−05	3.41E−03	6.09E−07	*EDN2*	*RNA5SP45*	
rs145738725	15:25826461	A/G	0.01304	14.46 (8.49~20.42)	2.50E−04	1.44E−03	2.06E−06		*LOC105370737*	*ATP10A*
rs77948500	12:60298988	C/G	0.01074	− 15.85 (− 22.39~− 9.31)	1.82E−06	2.29E−01	2.06E−06		*LOC100996696*	*PGBD3P1*
rs148501690	6:87012360	A/G	0.01437	− 13.88 (− 19.61~− 8.15)	2.33E−03	5.65E−05	2.06E−06		*NDUFA5P9*	*LOC100652960*
rs4234482	3:139844245	C/G	0.26610	− 3.64 (− 5.15~− 2.14)	6.24E−04	4.11E−04	2.16E−06	*CLSTN2*	*NMNAT3*	*LOC105374132*
rs74400620	7:121002702	A/T	0.01364	13.98 (8.13~19.8)	1.61E−06	8.51E−01	2.83E−06	*FAM3C*	*WNT16*	*CYCSP19*
rs2071336	16:12061674	A/G	0.03253	8.82 (5.04~12.59)	2.41E−05	8.37E−02	4.72E−06	*TNFRSF17*	*LOC729978*	*SNX29*
rs76054107	5:26671351	T/C	0.02113	10.76 (6.09~15.42)	1.39E−04	1.27E−02	6.11E−06		*MSNP1*	*CCNB3P1*
rs16835559	3:130749602	A/C	0.01467	12.47 (6.96~17.98)	1.48E−05	2.01E−01	9.19E−06	*NEK11*	*ASTE1*	*LOC339874*
rs1940556	14:88661112	T/C	0.01916	− 10.95 (− 15.80~− 6.10)	9.94E−05	3.62E−02	9.58E−06	*KCNK10*	*LOC105370612*	*SPATA7*

We failed to replicate the previously reported association between Thr164Ile (rs1800888, OR = 0.268, *P* = 0.712) and Gly16Arg (rs1042713, OR = 0.786, *P* = 0.7393) polymorphisms in the *ADRB2* and phenylephrine response [9, 10]. We also looked into all SNPs within ± 50 kb flanking adrenergic receptor genes, *ADRA1A*, *ADRA1B*, and *ADRB2*. Only 8 SNPs in an intron of *ADRA1A* showed a nominal significant association (*P* < 0.05) after meta-analyses (Additional file 4: Table S2).

### Further investigation of the top 3 lead SNPs in differentiation of subgroups of patients identified by *k*-means clustering

We performed association tests using binary traits by comparing the resistant group versus the sensitive group alone or the pooled sensitive + intermediate groups. We also evaluated the interaction of SNPs with body weight by adding an interaction term in the regression models. Results are summarized in Table 4. Only the genotype of rs11572377 in *END2* significantly associated with the resistant group in comparison with resistant vs. sensitive groups (*P* = 0.0153 and 0.0184) and resistant versus sensitive + intermediate groups (*P* = 0.047 and 0.0182) in both phase I and phase II cohorts. The regional association plot for *EDN2* and the boxplot for infusion rates by rs11572377 genotypes are shown in Fig. 4c and d, respectively, at an additive mode of inheritance. rs2069661 was found significant only in the phase I but not in the phase II cohort in both resistant versus sensitive or resistant versus combined sensitive + intermediate groups. rs77080086 did not show any significance across comparisons, suggesting that its association with phenylephrine infusion rate may be confounded by other factors.

**Table 4:** Summary of the results for the top 3 variants from the association testing

SNP	Average infusion rate				Resistant vs. sensitive				Resistant vs. (sensitive + intermediate)			
	Phase I (*N* = 1205)		Phase II (*N* = 329)		Phase I (*N* = 589)		Phase II (*N* = 147)		Phase I (*N* = 1205)		Phase II (*N* = 329)	
	*β*	*P*	*β*	*P*	OR	*P*	OR	*P*	OR	*P*	OR	*P*
rs2069661 *(F2RL2)*	13.71 (7.81~19.6)	5.71E−06	14.32 (3.28~25.4)	0.012	3.50 (1.52~8.05)	3.19E−03	1.84 (0.26~13.18)	0.545	3.90 (1.97~7.70)	9.12E−05	2.28 (0.43~12.22)	0.335
Sex	− 4.05 (− 6.34~− 1.76)	5.44E−04	1.44 (− 2.87~5.74)	0.513	0.50 (0.33~0.75)	7.97E−04	0.59 (0.25~1.35)	0.210	0.71 (0.50~1.03)	0.068	1.00 (0.48~2.08)	0.995
Age	0.08 (− 0.01~0.17)	0.078	0.16 (− 0.01~0.32)	0.065	0.97 (0.96~0.99)	2.58E−03	0.99 (0.95~1.02)	0.429	1.01 (0.99~1.02)	0.335	1.00 (0.97~1.03)	0.974
Body weight	0.14 (0.09~0.19)	6.84E−08	0.18 (0.09~0.27)	1.19E−04	1.01 (1.00~1.02)	0.019	1.02 (0.99~1.04)	0.139	1.01 (1.01~1.02)	4.40E−04	1.01 (0.997~1.03)	0.112
SNPxWeight	0.33 (0.08~0.57)	9.26E−03	0.05 (− 0.75~0.85)	0.895	1.01 (0.98~1.05)	0.512	1.04 (0.90~1.21)	0.581	1.02 (0.99~1.06)	0.172	1.02 (0.91~1.15)	0.695
rs77080086 *(PDE4B)*	12.17 (7.43~16.91)	5.50E−07	6.43 (− 3.32~16.2)	0.197	1.40 (0.69~2.8)	0.35	1.59 (0.33~7.57)	0.562	1.75 (0.93~3.30)	0.082	1.7 (0.40~7.21)	0.468
Sex	− 3.81 (− 6.09~− 1.53)	1.08E−03	1.10 (− 3.24~5.45)	0.620	0.51 (0.34~0.77)	1.09E−03	0.55 (0.24~1.26)	0.155	0.74 (0.51~1.06)	0.098	0.95 (0.46~1.97)	0.899
Age	0.07 (− 0.02~0.02)	0.115	0.16 (− 0.01~0.33)	0.060	0.97 (0.96~0.99)	2.20E−03	0.99 (0.95~1.02)	0.459	1.01 (0.99~1.02)	0.387	1.00 (0.97~1.03)	0.943
Body weight	0.14 (0.09~0.19)	5.69E−08	0.18 (0.09~0.27)	8.74E−05	1.01 (1.00~1.02)	0.016	1.02 (0.996~1.04)	0.125	1.01 (1.01~1.02)	4.42E−04	1.01 (0.998~1.03)	0.096
SNPxWeight	− 0.05 (− 0.26~0.17)	0.679	0.36 (− 0.40~1.11)	0.354	0.99 (0.96~1.02)	0.641	0.99 (0.90~1.09)	0.832	1.00 (0.98~1.03)	0.953	1.00 (0.91~1.11)	0.995
rs11572377 *(EDN2)*	12.26 (6.32~18.2)	5.44E−05	15.14 (5.08~25.19)	3.41E−03	3.95 (1.30~11.97)	0.015	5.97 (1.35~26.32)	0.018	2.03 (0.97~4.25)	0.047	4.3 (1.28~14.63)	0.018
Sex	− 4.05 (− 6.32~− 1.78)	4.86E−04	0.88 (− 3.40~5.17)	0.687	0.52 (0.35~0.78)	1.72E−03	0.54 (0.23~1.26)	0.151	0.72 (0.50~1.04)	0.079	0.9 (0.45~1.94)	0.848
Age	0.078 (− 0.01~0.17)	0.086	0.17 (0.01~0.03)	0.038	0.97 (0.96~0.99)	4.42E−03	1.00 (0.96~1.02)	0.526	1.01 (0.99~1.02)	0.335	1.0 (0.97~1.03)	0.862
Body weight	0.13 (0.08~0.18)	3.28E−07	0.18 (0.09~0.27)	1.32E−04	1.01 (1.00~1.02)	0.021	1.02 (0.998~1.04)	0.068	1.01 (1.01~1.02)	7.83E−04	1.0 (0.997~1.03)	0.110
SNPxWeight	0.01 (− 0.22~0.24)	0.96	− 0.26 (− 0.61~0.10)	0.155	1.05 (0.97~1.12)	0.219	0.97 (0.88~1.06)	0.479	1.01 (0.98~1.03)	0.613	0.96 (0.91~1.01)	0.120

The effect sizes for SNP, sex, age, and body weight were the main effect size in linear or logistic regression models for the quantitative or categorical traits respectively. The effect size of SNP × Weight was the effects of the interaction terms in the models which include only the SNP, body weight, and their interaction. Regression *β* and odds ratio (OR) were presented as mean (95% confidence interval)

There was no significant interaction between the genotype of rs11572377 and confounding factors such as body weight (*P* > 0.05), age (data not shown), and sex (data not shown), suggesting that rs11572377 is an independent genetic factor associated with phenylephrine response.

We searched STRING to illustrate interactive proteins for EDN2. The high-confidence interactive proteins included ADRA1B and ADRA1D, which are the targets of phenylephrine (Fig. 4e).

## Discussion

In this study, we leveraged comprehensive EHR data from Geisinger and applied an unsupervised machine learning approach to classify patients who had quantifiable phenylephrine infusion rates during surgery into three subcategories: resistant (high infusion with rate low BP), intermediate (low infusion rate with low BP), and sensitive (low infusion rate with high BP). Comorbidity hierarchical clustering showed the resistant group had a higher prevalence of confounding factors including heart failure, chronic kidney diseases, and acid-base imbalance, and were distinct from the intermediate and sensitive groups. Meta-analysis of the summary statistics from the phase I and phase II GWAS identified 12 independent loci with *P* < 10^−5^ in meta-analysis for infusion rates (Table 3). We further tested the association of the top three hits (*P* < 1 × 10^−6^) in the three subgroups identified by *k*-means clustering. Only rs11572377 at the 3′UTR of *EDN2* was significantly different in both the extreme cases (sensitive vs resistant) and all cases (resistant vs sensitive + intermediate) in phase I and phase II cohorts (*P* < 0.05). There is no significant interaction between this genotype and confounding factors such as body weight, age, or sex, suggesting that rs11572377 is an independent genetic factor associated with phenylephrine response.

*EDN2* encodes endothelin-2, a secretory vasoconstrictive peptide that can cause potent long-lasting vasoconstriction by binding to ET_A_ receptors on arterial smooth muscle cells. Genetic polymorphisms of *EDN2* quantitatively associated with pretreatment DBP in hypertensives but not in normotensive individuals [26, 27]. Several signal-transduction pathways including NADPH-oxidases, phospholipases, Rho-kinase (RhoK), and cellular influx of calcium ions can be stimulated after activation of ET_A_ receptors [28–30]. Different molecular mechanisms are implicated in the initiation and maintenance of vasoconstrictor response toward several vasoconstrictor agonists [31–33]. Although endothelin-2 has only two amino acids difference from endothelin-1 and shows same affinity for ET_A_ and ET_B_ receptors as endothelin-1, it exhibits a distinct mechanism and pathway affinity for vasoconstriction [25, 34]. Further search of the PPI network using STRING identified two highly confident interaction proteins for EDN2 (confidence score ≥ 0.9): ADRA1B and ADRA1D, which are the targets of phenylephrine (Fig. 4e). There are 3 alpha-1-AR subtypes: ADRA1A, ADRA1B, and ADRA1D, all of which signal through the Gq/11 family of G-proteins. Nuclear ADRA1A-ADRA1B heterooligomers regulate phenylephrine-stimulated ERK signaling in cardiac myocytes. ADRA1D can also be stimulated by phenylephrine although to a lesser degree due to a much longer N-terminal domain than ADRA1A and ADRA1B [35].

There are some limitations to the study. First, it would have been preferable to use bolus injections of phenylephrine for analysis, as this represents a more usual clinical scenario, and the transient BP response is often clearly evident in routine anesthesia records. However, the available de-identified clinical data derived from anesthesia records did not incorporate all of the expected BP data. The blood pressures in the de-identified research dataset were not reliably frequent enough to assess blood pressure responses to phenylephrine boluses. Thus, infusion rates were used as a surrogate for assessment of phenylephrine sensitivity phenotypes since continuous infusions would span longer time intervals between BP values. Compared to previous candidate gene studies assessing phenylephrine sensitivity using the linear variable differential transformer dorsal hand vein technique [9, 12, 36], phenylephrine infusion rate represents a more clinically relevant approach to responsiveness. Also, the response displayed a continuous spectrum and no clear separation due to the complex nature of phenylephrine response. However, it may serve as a starting point for such studies of phenylephrine and may shed light on clinical insight.

Second, this study incorporated data from the entire range of anesthetics for all varieties of surgical procedures; many potentially confounding variations of patient condition and surgical requirements are unidentified and assumed to be randomly distributed across the clusters. Phenylephrine infusion rate could be potentially influenced by many nongenetic factors during anesthesia which could not be accounted for in the analyses. These include episodic blood loss, intravenous fluid boluses versus maintenance infusions, stimuli from surgery, long-term comorbidities, and pre or concurrent medication such as angiotensin converting enzyme inhibitors (ACEI). This could explain in part the observation that 97 patients who had two or more surgical episodes had inconsistent cluster assignment. The number of patients was insufficient to explore this hypothesis, but analysis of this subgroup could be used to explore potential gene by environment interactions impacting response to phenylephrine. Our preliminary analyses showed that premedication with ACEI or angiotensin II receptor blockers as a covariate have no significant impact (*P* = 0.258) on the association between *EDN2* SNPs and phenylephrine infusion rate after adding the interactive term (rs11572377 × drug) in the linear regression model.

Third, limitation to cases with phenylephrine infusions curtailed the number of cases available for analysis. We removed approximately 70% of the cases initially eligible in this study to minimize the effects of heterogeneity in the patient population and obvious major confounding factors. This additional filtering process improved homogeneity, but at the expense of a significant decrease in sample size. Analysis of genomic associations limited to cases having phenylephrine infusions is potentially biased by selection of records for more acutely compromised clinical cases with variations in tendency to hypotension under volatile general anesthetics and a wide variety of surgical procedures. Vasopressor infusions tend to be used when other interventions such as intravenous volume infusion are inadequate to maintain correction of hypotension. However, the approach to select extreme phenotypes may identify genetic factors with bigger effect size, thus increasing statistical power. To quantify the potential impact of the decreased sample size, we conducted a power test using Quanto, given the main effect of β_G_ (around 13 for rs11572377 from the meta-analysis), a type 1 error rate of 1 × 10^−4^ for a suggestive significance with a two-sided test, on the continuous trait with mean ± SD of average infusion rate as 37.33 ± 19.7. Our sample size of 1534 from phase I and phase II samples had more than 80% power to identify a significant association when the minor allele frequency was equal to 0.017. Genotyping and exome sequencing of additional consented participants will increase size of potential analytic cohorts. With larger cohorts, the opportunity to select more homogeneous groups for genetic analysis may resolve issues confounding this preliminary investigation.

Fourth, phenylephrine infusion is frequently used to treat hypotension induced by spinal anesthesia in cesarean section [37, 38]. We excluded this group of patients because the indication and procedure are significantly different than general anesthesia and because of the prior observation that phenylephrine response in this group could be different [39, 40]. Future studies could include general or spinal anesthesia as distinct categories.

This preliminary investigation has exposed numerous challenges and opportunities to improve the extraction of appropriate characteristics from routine clinical EHR, so that functional phenotypes can be better defined and distinguished as necessary adjuncts for genomic analysis. Improving extraction of details available in routine anesthesia records will greatly improve definition of functional phenotypes for future studies, likely to increase events available for analysis by an order of magnitude. Aggregating data from numerous institutions has been a major challenge of the Multi-Center Perioperative Outcomes Group (MPOG) due to differences in participating organization practices and data formats [41–43]. Yet, that effort has been rewarded by opportunities to study rare events by increasing the number of cases available in the denominator [44]. Similar approaches will enhance the opportunity to understand genomic factors for populations and individuals as genomic data become more readily available, emphasizing the importance of preliminary studies that can develop broadly applicable methods to promote data sharing and power new knowledge discovery.

## Conclusions

In this study, we described a novel strategy to analyze “real-world” EHR data followed by GWAS to identify genetic factors associated with phenylephrine infusion rate during anesthesia. Through *k*-means clustering, we identified three subgroups of patients who were “resistant,” “intermediate,” and “sensitive” to phenylephrine infusion. Through meta-analyses of the phase I and phase II GWAS, we identified rs11572377, a 3′UTR variant of *EDN2*, as one of the top hits associated with differential response to phenylephrine infusion rate. This study demonstrated the EHR data can be a powerful resource for anesthesiology research. Future studies with more detailed extraction of data from clinical anesthesia records and other available clinical data will help to improve phenomic characterization for research and help with understanding of phenylephrine response.

## Additional files

Additional file 1: **Table S1.** The association of potential covariates with average infusion rates using a linear model. (DOCX 13 kb)

Additional file 2: **Figure S1.** *k-*means clustering based on phenylephrine infusion rate, mean and SD values of SBP when *k* = 2. (DOCX 50 kb)

Additional file 3: **Figure S2.** Evaluating *k*-means clustering on different feature combinations (DOCX 418 kb)

Additional file 4: **Table S2.** Summary of SNPs in ADRA1A that showed nominal association (*P* < 0.05) with phenylephrine infusion rate. (DOCX 16 kb)

## Data Availability

The GWAS summary result generated in this study are available from the corresponding author on request. The individual EHR and genetics datasets (even de-identified) used and/or analyzed during the current study are not publicly available due to Geisinger Policy and the term with Regeneron Genetics Center contract. Collaboration requests and data use agreements with Geisinger are necessary to obtain access to the deidentified EHR data.

## Abbreviations

EDN2: Endothelin-2
EHR: Electronic health record
ET_A_: Endothelin type A
GWAS: Genome-wide association study
ICD-9: International Classification of Disease v. 9
IV: Intravenous
LD: Linkage disequilibrium
MyCode: MyCode® Community Health Initiative
PC: Principal component
SBP: Systolic blood pressure
SD: Standard deviation
SNP: Single nucleotide polymorphisms
