# Supplementary material for: Dissecting genetic factors affecting phenylephrine infusion rates during anesthesia: a genome-wide association study employing EHR data
Source: BMC Med. 2019 Aug 28;17:168. doi: 10.1186/s12916-019-1405-7 (PMC6712853; doi:10.1186/s12916-019-1405-7)
Supplement: Supplementary file 1 — Table S1. The association of potential covariates with average infusion rates using a linear model. (DOCX 13 kb) [file 12916_2019_1405_MOESM1_ESM.docx]

Table S1. The association of potential covariates with average infusion rates using a linear model.

| **Estimate** | **Estimate** | **SE** | **t-value** | **P** | **Significance** |
| --- | --- | --- | --- | --- | --- |
| Age at the surgery | 0.096 | 0.041 | 2.341 | 0.019 | * |
| Mean SBP | -0.307 | 0.043 | -7.101 | 1.90E-12 | *** |
| SD of SBP | 0.537 | 0.075 | 7.170 | 1.17E-12 | *** |
| Body weight (kg) | 0.133 | 0.024 | 5.665 | 1.76E-08 | *** |
| Female sex | -3.050 | 1.059 | -2.879 | 0.004 | ** |
| General anesthesia | -2.916 | 1.710 | -1.705 | 0.088 |  |
| Spinal anesthesia | -0.535 | 3.848 | -0.139 | 0.889 |  |
| 250: Diabetes | 1.690 | 1.697 | 0.995 | 0.320 |  |
| 272: Lipoid metabolism disorders | 0.095 | 1.686 | 0.057 | 0.955 |  |
| 278: Overweight and obesity | 1.249 | 2.093 | 0.597 | 0.551 |  |
| 401: Hypertension | 2.327 | 1.862 | 1.250 | 0.211 |  |
| 414: Other forms of chronic ischemic heart diseases | -2.384 | 1.828 | -1.304 | 0.192 |  |
| 427: Cardiac dysrhythmias | 1.838 | 2.163 | 0.850 | 0.396 |  |
| 428: Heart Failure | 1.602 | 2.120 | 0.755 | 0.450 |  |

Full linear model was adopted to include all covariates without interaction terms. Only the variables which could significantly explain the variation of average infusion rate were included as covariates in the subsequent SNP association testing.
