# Supplementary material for: Dissecting genetic factors affecting phenylephrine infusion rates during anesthesia: a genome-wide association study employing EHR data
Source: BMC Med. 2019 Aug 28;17:168. doi: 10.1186/s12916-019-1405-7 (PMC6712853; doi:10.1186/s12916-019-1405-7)
Supplement: Supplementary file 2 — Figure S1. k-means clustering based on phenylephrine infusion rate, mean and SD values of SBP when k = 2. (DOCX 50 kb) [file 12916_2019_1405_MOESM2_ESM.docx]

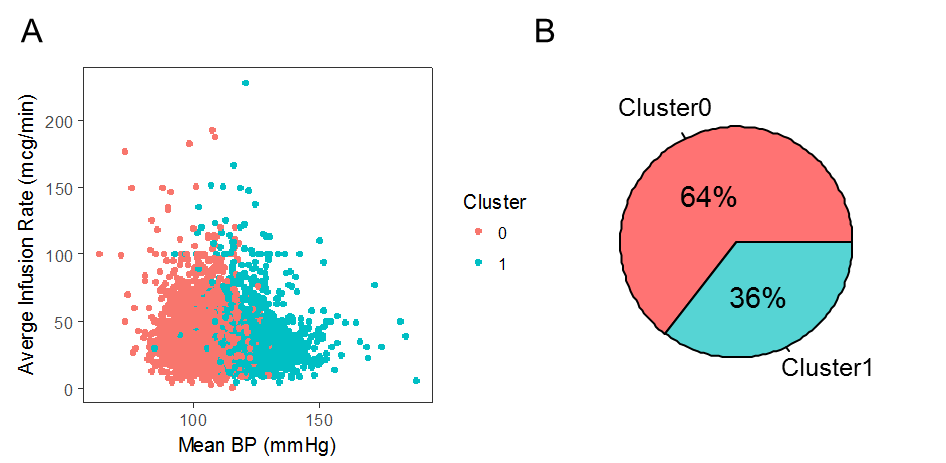


Fig. S1. K-means clustering based on phenylephrine infusion rate, mean and SD values of SBP when k=2. (A) Scatter plot of mean SBP against phenylephrine average infusion rate. (B) Pie chart to show the proportion of identified each subgroup. SBP: systolic blood pressure
