# Supplementary material for: Dissecting genetic factors affecting phenylephrine infusion rates during anesthesia: a genome-wide association study employing EHR data
Source: BMC Med. 2019 Aug 28;17:168. doi: 10.1186/s12916-019-1405-7 (PMC6712853; doi:10.1186/s12916-019-1405-7)
Supplement: Supplementary file 3 — Figure S2. Evaluating k-means clustering on different feature combinations (DOCX 418 kb) [file 12916_2019_1405_MOESM3_ESM.docx]

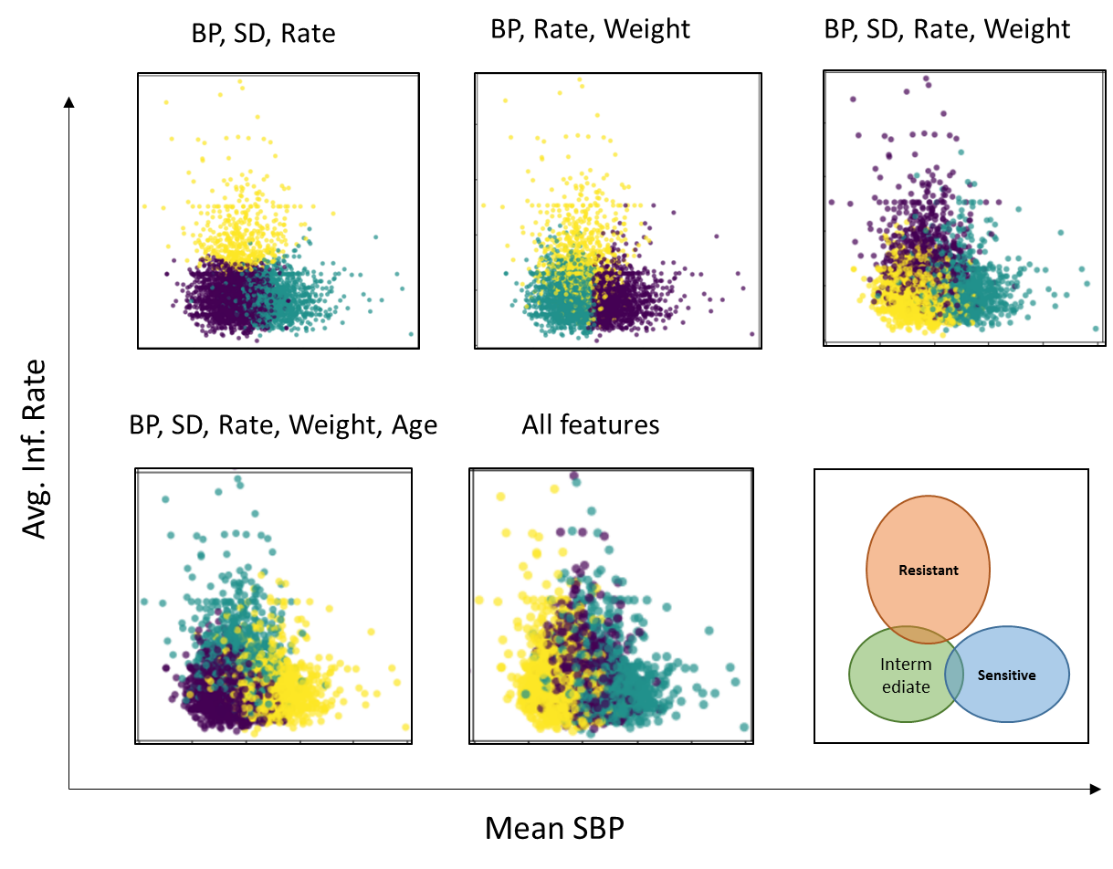


Fig.S2: Evaluating K-means clustering on different feature combinations. K-mean clustering was performed on different feature combinations including mean SBP, SD of SBP, average infusion rate, body weight, age at surgery, and infusion duration. To visualize the subgroups with different responsiveness, we plot the Mean SBP on X-axis and average infusion rate on Y-axis.
