# Supplementary material for: Dissecting genetic factors affecting phenylephrine infusion rates during anesthesia: a genome-wide association study employing EHR data
Source: BMC Med. 2019 Aug 28;17:168. doi: 10.1186/s12916-019-1405-7 (PMC6712853; doi:10.1186/s12916-019-1405-7)
Supplement: Supplementary file 4 — Table S2. Summary of SNPs in ADRA1A that showed nominal association (P < 0.05) with phenylephrine infusion rate. (DOCX 16 kb) [file 12916_2019_1405_MOESM4_ESM.docx]

Table S2. Summary of SNPs in *ADRA1A* that showed nominal association (p < 0.05) with phenylephrine infusion rate.

| **SNP** | **Coordinate(HG19)** | **A1** | **A2** | **Beta** | **L95** | **U95** | **P-Dis** | **P-Rep** | **P-meta** |
| --- | --- | --- | --- | --- | --- | --- | --- | --- | --- |
| rs35462196 | 8:26667707 | A | C | -6.82 | -12.09 | -1.55 | 0.031 | 0.372 | 0.011 |
| rs6557949 | 8:26647989 | T | G | 6.06 | 0.90 | 11.23 | 0.031 | 0.187 | 0.021 |
| rs7839120 | 8:26659511 | A | G | -6.81 | -12.09 | -1.54 | 0.031 | 0.187 | 0.011 |
| rs34996142 | 8:26668783 | T | C | 6.81 | 1.54 | 12.08 | 0.030 | 0.187 | 0.011 |
| rs12541572 | 8:26708986 | T | C | -2.14 | -4.16 | -0.11 | 0.215 | 0.033 | 0.039 |
| rs80033897 | 8:26707785 | T | C | -2.13 | -4.16 | -0.11 | 0.215 | 0.033 | 0.039 |
| rs13265045 | 8:26670896 | T | C | 7.22 | 1.80 | 12.64 | 0.025 | 0.187 | 0.009 |
| rs13248896 | 8:26643191 | A | G | 6.06 | 0.90 | 11.23 | 0.031 | 0.372 | 0.021 |
| rs13275271 | 8:26641824 | T | C | 6.06 | 0.90 | 11.23 | 0.031 | 0.372 | 0.021 |
| rs7017511 | 8:26642410 | T | C | -6.06 | -11.23 | -0.90 | 0.031 | 0.372 | 0.021 |
| rs13276482 | 8:26656653 | T | C | 6.48 | 1.26 | 11.70 | 0.031 | 0.257 | 0.015 |
| rs11991324 | 8:26651694 | A | C | -6.48 | -11.70 | -1.26 | 0.031 | 0.257 | 0.015 |
